# Dependence of Sensitivity, Derivative of Transfer Curve and Current on Bias Voltage Magnitude and Polarity in Tunneling Magnetoresistance Sensors

**DOI:** 10.3390/s23031214

**Published:** 2023-01-20

**Authors:** Łukasz Fuśnik, Bartłomiej Szafraniak, Jerzy Wrona, Susana Cardoso, Paulo. P. Freitas, Piotr Wiśniowski

**Affiliations:** 1 Institute of Electronics, AGH University of Science and Technology, 30-059 Krakow, Poland; 2 Singulus Technologies AG, 63796 Kahl am Main, Germany; 3 INESC Microsystems and Nanotechnologies, INESC-MN, and IN, 1000-029 Lisbon, Portugal; 4 Physics Department, Instituto Superior Tecnico, Universidade de Lisboa, 1600-276 Lisbon, Portugal

**Keywords:** magnetic field sensors, tunneling magnetoresistance sensors, sensing characteristics, bias voltage effect on sensing characteristics, voltage controlled magnetic anisotropy (VCMA)

## Abstract

The sensitivity of tunneling magnetoresistance sensors is an important performance parameter. It depends on the derivative of resistance versus magnetic field (transfer curve) and the current and is expressed as the product of the two factors. Previous research has demonstrated that the bias voltage has a significant impact on the sensitivity. However, no research has been conducted into the dependence of current and the derivative on bias voltage magnitude and polarity, and their contribution to the sensitivity. Thus, this paper investigates the dependence of sensitivity, derivative of resistance versus magnetic field curve and current on bias voltage magnitude and polarity in CoFeB/MgO/CoFeB-based tunneling magnetoresistance sensors with weak, strong and no voltage-controlled perpendicular magnetic anisotropy modification. It demonstrates that the sensitivity dependence on bias voltage for sensors with voltage controlled magnetic anisotropy modification showed no saturation up to 1 V. Moreover, the sensitivity asymmetry with respect to bias polarity changed significantly with bias, reaching a ratio of 6.7. Importantly, the contribution of current and the derivative of resistance versus magnetic field curve to the sensitivity showed a crossover. The current dominated the bias dependence of sensitivity below the crossover voltage and the derivative above the voltage. Furthermore, the crossover voltage in sensors without voltage controlled magnetic anisotropy modification did not depend on polarity, whereas in sensors with voltage controlled magnetic anisotropy modification, it appeared at significantly higher voltage under positive than negative polarity.

## 1. Introduction

Tunneling magnetoresistance (TMR) sensors [1] with CoFeB-MgO-CoFeB materials structure have several characteristics that are attractive for high performance magnetic field sensing. They can be designed with arbitrary resistance by tuning tunneling barrier thickness and size. This enables the design of sensors with desirable resistance and ultra-low power consumption. The sensors can be fabricated to have dimensions down to nm thus enabling magnetic field sensing with high spatial resolution. Moreover, TMR sensors with the perpendicular anisotropy in the sensing layer [2] offer simple and effective modification [3] of sensing range and sensitivity by thickness-induced the anisotropy modulation and improvement of the magnetic field detection level below the magnetic noise-sensitivity scaling limit [4].

Among these features and characteristics, the sensitivity is one of the key performance parameters of sensors. The strong sensitivity change with bias voltage is one of the distinct features of TMR sensors. The sensitivity (S) is typically calculated as a product of derivative (dR/dμ_0_H) of resistance versus magnetic field curve (R-H) and current (I), i.e., S = I × dR/dμ_0_H. Due to tunneling, the current significantly changes with bias [5,6] thus the sensitivity does. As a result, for a typical TMR sensor, the sensitivity dependence on bias shows three characteristics [7]. It sharply increases at low bias voltage. At a certain voltage, it reaches a maximum and then decreases. Moreover, it is not significantly affected by the bias polarity [7]. Importantly, the sensitivity also affects the magnetic noise [4] and field detection [7]. All this implies that the bias voltage dependence of the sensitivity is an important characteristic for applications and design of the TMR sensors.

It has been observed, however, that for TMR sensors with the voltage controlled magnetic anisotropy (VCMA) effect [8,9], the sensitivity is significantly affected not only by the bias voltage magnitude but also by the bias polarity. We showed that the sensitivity can be significantly modified by reversing bias [10]. The reversal resulted in an up factor of two changes in the sensitivity at maximal bias magnitude. Further investigation [11] of the influence of bias polarity on sensitivity confirmed a factor of two changes in the sensitivity upon voltage polarity reversal. This study indicated the possibility of control and modification of the sensitivity by the bias polarity in sensors with VCMA.

Furthermore, we investigated the sensitivity dependence on bias voltage for sensors without [7] and with VCMA [11] modification. Because sensitivity is the product of current and the derivative of R-H, both of which are strongly influenced by bias magnitude [7,12] and polarity [10,11] in TMR sensors, it is important to analyze and evaluate their contributions to the dependence of sensitivity on bias magnitude and polarity. However, no study of the dependence of current and derivative of resistance versus magnetic field curve on bias voltage magnitude and polarity for TMR sensors with or without VCMA modification has been conducted.

Therefore, this work investigates the dependence of sensitivity (S = dR/dμ_0_H × I), derivative (dR/dμ_0_H) of resistance versus magnetic field curve (R-H) and current (I) on bias voltage magnitude and polarity in CoFeB/MgO/CoFeB-based TMR sensors with weak, strong and no measurable VCMA modification. It shows that the sensitivity dependency on bias voltage for sensors with VCMA modification showed features that have not been observed in TMR sensors. At a bias polarity that reduces the anisotropy, the sensitivity increases sharply and does not reach saturation up to 1 V. The sensitivity asymmetry with respect to the bias polarity changed strongly with bias and reached a ratio as high as 6.71. Importantly, the contribution of current and derivative of R-H to the sensitivity showed a crossover. In all sensors, current dominated the bias dependence of sensitivity below the crossover voltage and above the derivative of R-H. Furthermore, the crossover voltage in sensors without VCMA did not depend on polarity, whereas in sensors with VCMA, it appeared at a significantly higher voltage under positive polarity than negative polarity.

## 2. Fabrication and Measurements of Sensors

**Figure 1:**
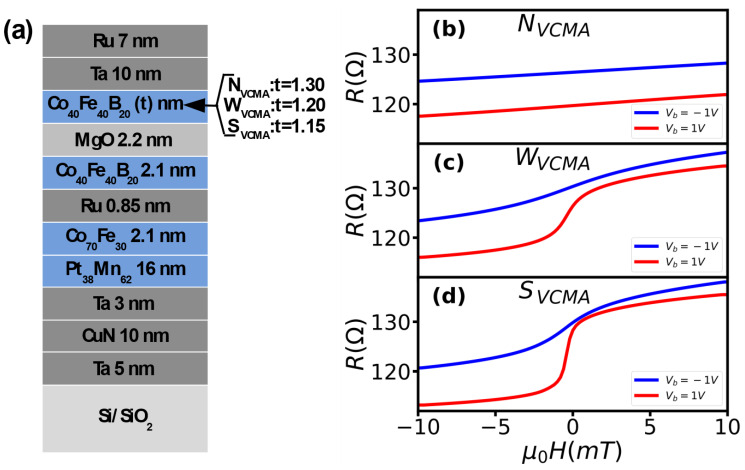
Material structure of the sensors (**a**), transfer curves of sensors without (**b**) and with VCMA modification (**c**,**d**) for positive and negative bias voltage.

To study the bias voltage dependence of sensitivity, we fabricated sensors with varying (wedge) thicknesses (t) of the sensing layer from 1.15 nm to 1.35 nm and materials stack SiO_2_/Ta(5)/CuN(10)/Ta(3)/PtMn_62_(16)/CoFe_30_(2.1)/Ru(0.85)/Co_40_Fe_40_B_20_ (2.1)/MgO(2.2)(RA ~62 kOhmμm^2^)/Co_40_Fe_40_B_20_(t)/Ta(10)/Ru(7), thickness in nanometers (Figure 1a). A range of sensing layer thicknesses was selected to obtain sensors with varying strength of perpendicular anisotropy (PA). The sensor material stack was deposited on a 4-in. wafer using a TIMARIS sputtering system at Singulus AG using a linear dynamic deposition technique. The metallic layers were deposited by DC magnetron sputtering and MgO by radio frequency magnetron sputtering. The linear dynamic deposition technique guarantees the homogeneity and thickness of the layers. The structure and thickness of the layers were verified during the optimization of the deposition process at Singulus AG. The 4-in. wafer was diced into 1 in. × 1 in. and die with the thickness of the sensing layer from t = 1.15 nm to 1.35 nm was selected for fabrication based on previous results [2]. The different sensing layer thicknesses enabled the fabrication of sensors with strong (t = 1.15), medium (t = 1.20) and weak (t = 1.30) perpendicular anisotropy that enables observation of different degrees of the PA modulation by bias voltage [13]. The sensors were fabricated at INESC-MN using a laser lithography microfabrication process. The 1-in. wafer was patterned using direct-writing laser lithography and ion beam milling. All sensors were patterned into circular shapes with a diameter of 30 µm. The patterned 1 × 1-in wafer was annealed in a high vacuum at 330 °C for 1 h in a magnetic field of 0.5 T.

To investigate the dependence, we measured resistance versus magnetic field curves (R-H). The R-H curves were measured using a DC two-probe setup consisting of Helmholtz coils as a magnetic field source and a source measure unit (Keithley 2400) to bias the sensors. The constant voltage source mode of the source measure unit was used to bias the sensors, and the resistance value was determined by measuring the corresponding current during the magnetic field sweep. The current was measured with a bias voltage ranging from +1 V to −1 V in 20 mV steps. We extracted current from R-H curves for zero bias magnetic field. The derivatives of R-H curves were computed by differentiation of R-H curves for zero bias magnetic field. The sensitivity was calculated as a product of current and derivative of R-H.

## 3. Results

### 3.1. Resistance versus Magnetic Field Curves

The sensors with the thinnest sensing layers (strong PA) show no measurable VCMA modification (N_VCMA_) of perpendicular anisotropy by bias polarity (Figure 1b). The bias polarity had no effect on the sensors’ resistance versus magnetic field curves (transfer curves), which is an indicator of the VCMA modification. Regardless of polarity, the R-H curves show linear dependence on the magnetic field and the same slope. This is typical for sensors with strong perpendicular anisotropy [14], which require high voltage (above breakdown) to modify the anisotropy, thus the appearance of the VCMA effect. For this sensor, the only measurable bias polarity influence was on its resistance, which varied up to 7.0 Ohm between +1 and −1 volts.

In contrast, the sensors with thicker sensing layers showed weak (W_VCMA_) and strong (S_VCMA_) VCMA modification, as indicated by changes in slope of the transfer curves (Figure 1c,d). For the sensor with medium thickness of the sensing layer, the slope of the transfer curves changed from 1.14 Ohm/mT at −1 V to 4.69 Ohm/mT at +1 V (Figure 1c). The sensor with the thicker sensing layer showed the change in the slope between positive and negative bias as high as 8.09 Ohm/mT (10.4 Ohm/mT at +1 V 2.31 Ohm/mT at −1 V) (Figure 1d). The change in the slopes upon bias reversal indicates the presence of VCMA modification in both sensors. Whereas the different slope values in the sensor indicate varying degrees of perpendicular anisotropy modification by the bias. The anisotropy strength determines the degree of modification, which is inversely proportional to the sensing layer thickness, i.e., the thinner the layer, the stronger the perpendicular anisotropy.

### 3.2. Sensitivity

**Figure 2:**
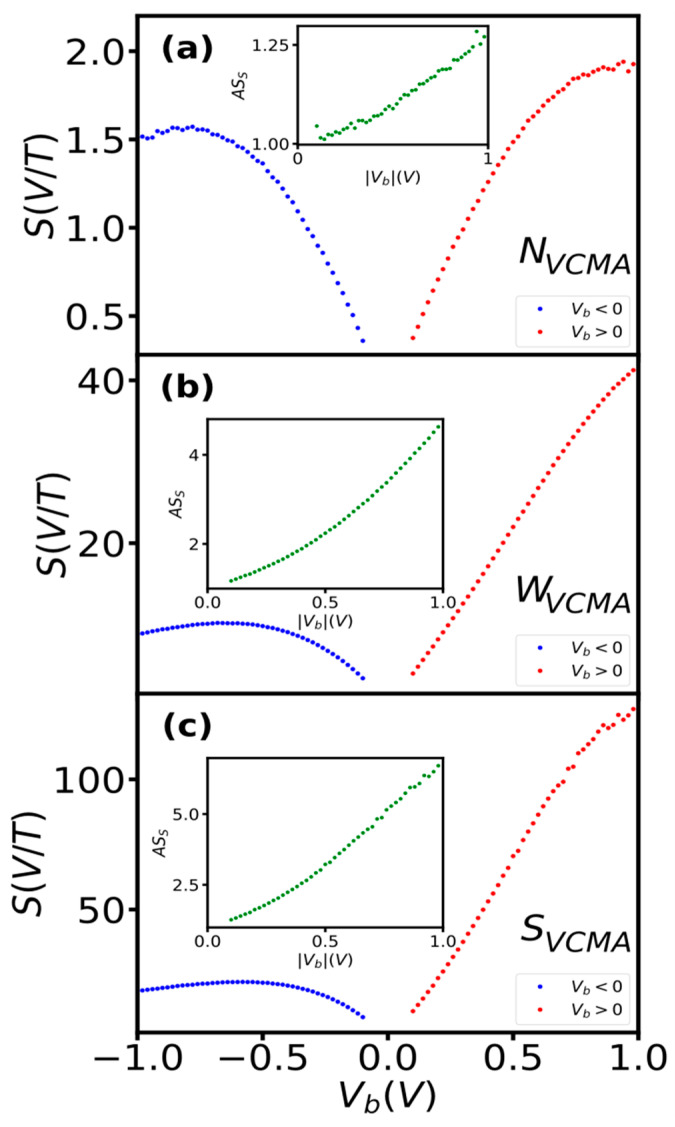
Bias voltage dependence of sensitivity of sensors without (**a**), with medium (**b**) and strong (**c**) VCMA modification. Inset the influence of bias polarity on symmetry of the sensitivity. The asymmetry was computed as AS_S_ = S(+V_b_)/S(−V_b_).

Sensors without measurable VCMA modification (N_VCMA_) showed the typical [7,12] dependence of sensitivity on bias voltage (Figure 2a). The sensitivity increased with bias in both polarities up to a certain voltage magnitude (here, approximately +/−750 mV) and reached a slightly higher value at positive (2 V/T) than at negative (1.6 V/T) voltage. The asymmetry (AS_S_) of the sensitivity with respect to bias polarity was low and did not exceed 1.28 (inset of Figure 2a). Finally, the sensitivity began to decrease above the saturation voltage. The sensor without the VCMA effect showed a change in the sensitivity with bias voltage magnitude and a slight polarity asymmetry, which is typical for TMR sensors [1,7,12].

For sensors that show weak (W_VCMA_) and strong (S_VCMA_) VCMA modification, the dependence of sensitivity on bias voltage showed new features that have not been observed in TMR sensors (Figure 2b,c). Under positive voltage, the sensitivity increases sharply and does not reach saturation up to 1 V (measured range). In contrast, at negative bias, it saturates fast and only slightly reduces over a significant range. The sensitivity reached a significantly larger value at positive bias (41.3 V/T, 127 V/T) than at negative bias (10.2, 22.2 V/T). The asymmetry of the sensitivity (AS_S_) changed strongly with bias (insets of Figure 2b,c) reaching 4.63 and 6.71 for W_VCMA_ and S_VCMA_ sensors, respectively. The large asymmetry in the sensitivity dependence on bias indicates a strong impact of the VCMA on the current and/or the derivative of R-H or both, as the sensitivity is a function of the current and the derivative of resistance with respect to the magnetic field. The impact is discussed in subsequent sections.

### 3.3. Current

**Figure 3:**
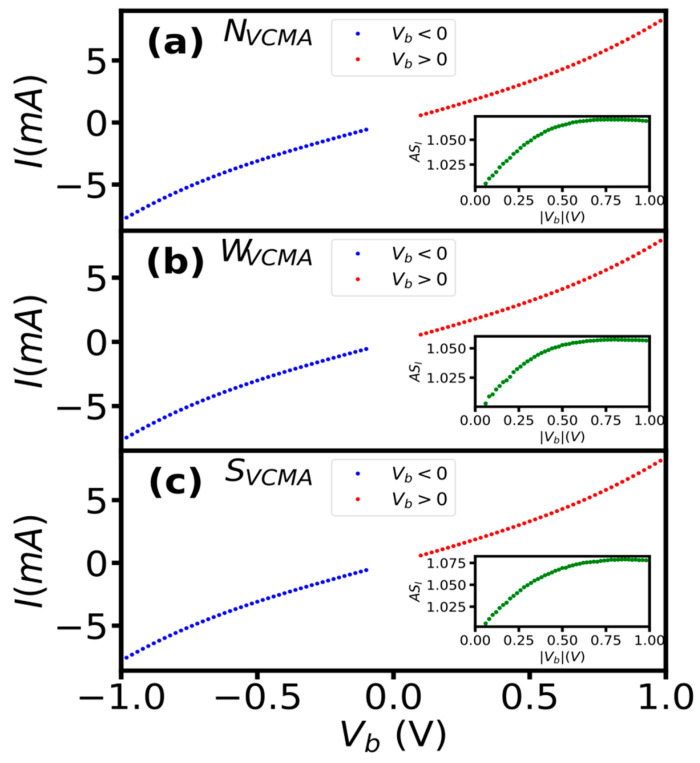
The influence of bias polarity on current and its symmetry (inset) of sensors without (**a**), with medium (**b**) and strong (**c**) VCMA modification. The asymmetry was computed as AS_I_ = I(+V_b_)/I(−V_b_).

All sensors showed approximately the same dependence of current on bias voltage for both polarities (Figure 3). We extracted current from R-H curves measured in the bias range +/−50 to +/−1 V to evaluate the effect of bias voltage on current. The shapes of I–V curves are very similar for sensors with and without VCMA modification (Figure 3). Moreover, it resembles typical dependence in tunneling regime devices [14,15]. The asymmetry of current (AS_I_) with respect to bias polarity curves was approximately the same for all sensors and did not exceed 1.08. The I–V characteristics showed negligible influence of the bias polarity (VCMA modification) on the current change with bias in all sensors.

**Figure 4:**
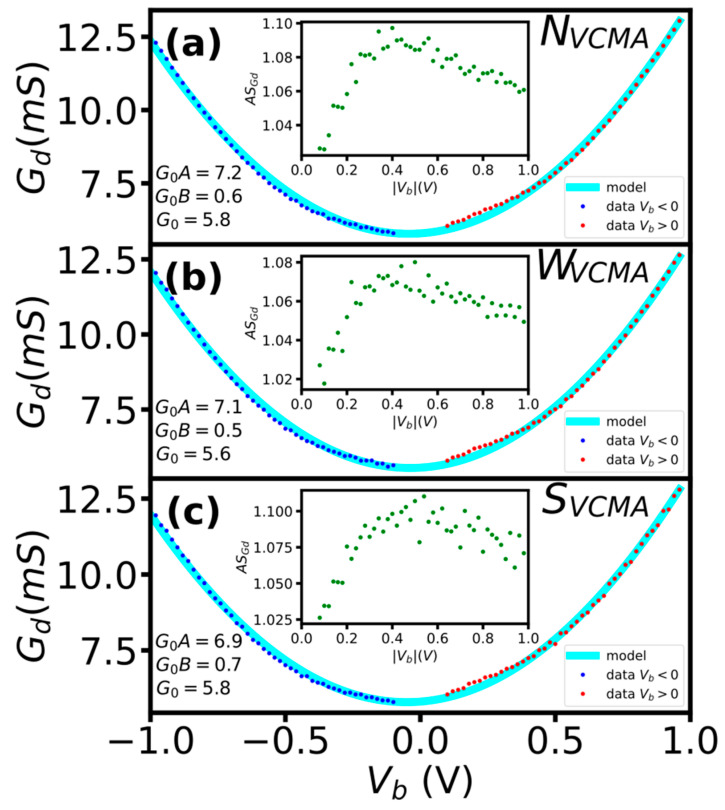
The influence of bias polarity on dI/dV characteristics and their symmetry (inset) of sensors without (**a**), with medium (**b**) and strong (**c**) VCMA modification. The asymmetry was computed as AS_Gd_ = G_d_(+V_b_)/G_d_(−V_b_).

The bias polarity also had no significant influence on the rate of current change (dynamic conductance) in sensors with and without the VCMA modification (Figure 4). To further evaluate the influence of bias voltage on the rate of current change, we computed the dynamic conductance G_d_ = dI/dV. The G_d_(V) curves for sensors with and without VCMA modification are very similar, resembling the quadratic shape typical of tunneling devices [15]. To quantify the influence of the bias polarity, we fitted the G_d_(V) curves with the conductance G_d_(V) = G_0_ + AG_o_V + BG_o_V^2^ model [14,15]. The fitting coefficients (A, B) were approximately the same, with A differing by no more than 6% and B by 4.2% (Figure 4). Moreover, we computed the asymmetry of the conductance (AS_Gd_) with respect to bias polarity curves for all sensors. The asymmetry was approximately the same and did not exceed 1.08. The G_d_(V) curve fitting parameters and the asymmetry show that the bias polarity had negligible influence on the rate of change in current in sensors, with and without the VCMA modification.

### 3.4. Derivative of Resistance versus Magnetic Field Curve

**Figure 5:**
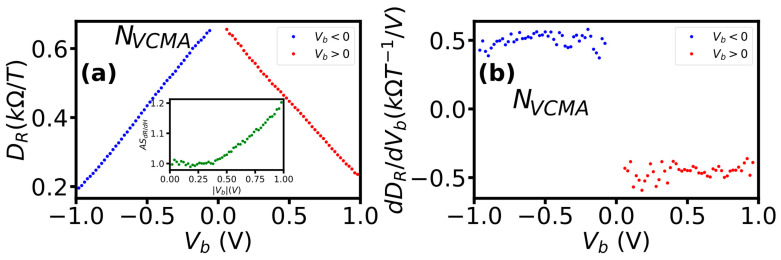
The influence of bias polarity on D_R_ = dR/dμ_0_H and its symmetry (inset) (**a**) and on the rate of D_R_s change (**b**) of sensors without VCMA modification. The asymmetry was computed as AS_dR/dH_ = dR/dμ_0_H(+V_b_)/dR/dμ_0_H(−V_b_).

**Figure 6:**
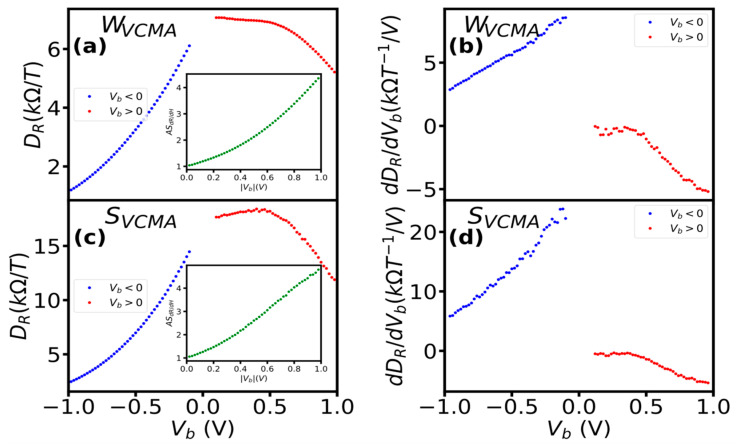
The influence of bias polarity on D_R_ = dR/dμ_0_H and their symmetry (inset) (**a**,**c**) and the rate of D_R_ (**b**,**d**) of sensors with weak and strong VCMA. The asymmetry was computed as AS_dR/dH_ = dR/dμ_0_H(+V_b_)/dR/dμ_0_H(−V_b_).

The sensor without VCMA modification (N_VCMA_) did not show a significant influence of bias polarity on both the derivative (D_R_ = dR/dμ_0_H) and its rate of change (dD_R_/dV) with the bias (Figure 5). The D_R_ decreased linearly with bias of both polarities and reached a slightly lower value at negative (0.189 kΩ/T,) than positive (0.235 kOhm/T) voltage (Figure 5a). For both polarities, the average rate of change of the derivative was about 0.45 kΩT^−1^/V (Figure 5b). The asymmetry of the derivative with respect to voltage polarity was low and did not exceed 1.19 (inset of Figure 6a). Moreover, the asymmetry appeared only for bias larger than 0.43 V. The monotonic, linear and at constant rate decrease in D_R_ with bias and its low asymmetry confirm that the sensors without VCMA modification showed typical (Figure 2a) for TMR sensors dependence of sensitivity on bias voltage.

The polarity of the bias voltage had a significant impact on the derivative and its rate of change with bias in sensors with the VCMA modification (Figure 6). At a negative bias, dR/dμ_0_H is reduced in the whole measured range. For sensor with weak VCMA, this resulted in a reduction of the derivative from 6.85 kΩ/T to 1.14 kΩ/T (Figure 6a) and for sensor with strong VCMA from 16.74 kΩ/T to 2.36 kΩ/T (Figure 6c). The rate of the dR/dμ_0_H reduction was approximately linear (Figure 6b). At positive bias, D_R_ showed different change depending on the bias range. In the bias range up to 0.5 V, it reduced by 0.14 kΩ/T with a constant rate of −0.38 for sensors with weak VCMA (Figure 6b) and increased by 0.97 kΩ/T with a constant rate of 1.52 for sensors with strong VCMA (Figure 6c). For bias above 0.5 V, the derivative decreased slightly (1.64 kΩ/T) for sensors with weak VCMA modification and substantially (5.95 kΩ/T) for sensors with strong VCMA modification. For both sensors, the rate of the dR/dμ_0_H reduction was approximately linear. The different dR/dμ_0_H changes with positive and negative bias, as evidenced by high asymmetry up to 5 (inset of Figure 6b,c), indicate a strong impact of the VCMA modification on dR/dμ_0_H bias dependence.

For sensors with and without the VCMA modification, both current and its rate of change (dI/dV) with bias (Figure 3 and Figure 4) show no influence of the bias polarity, thus the VCMA modification. In contrast, the derivative (dR/dμ_0_H) and its rate of change dependence on bias for sensors with VCMA modification differed strongly at positive and negative bias (Figure 6). This indicates that the derivative of R-H curves (dR/dμ_0_H) had a decisive influence on the observed features in bias voltage dependence of the sensitivity in sensors with VCMA modification.

### 3.5. Normalized Current and Derivative of R-H Curve

**Figure 7:**
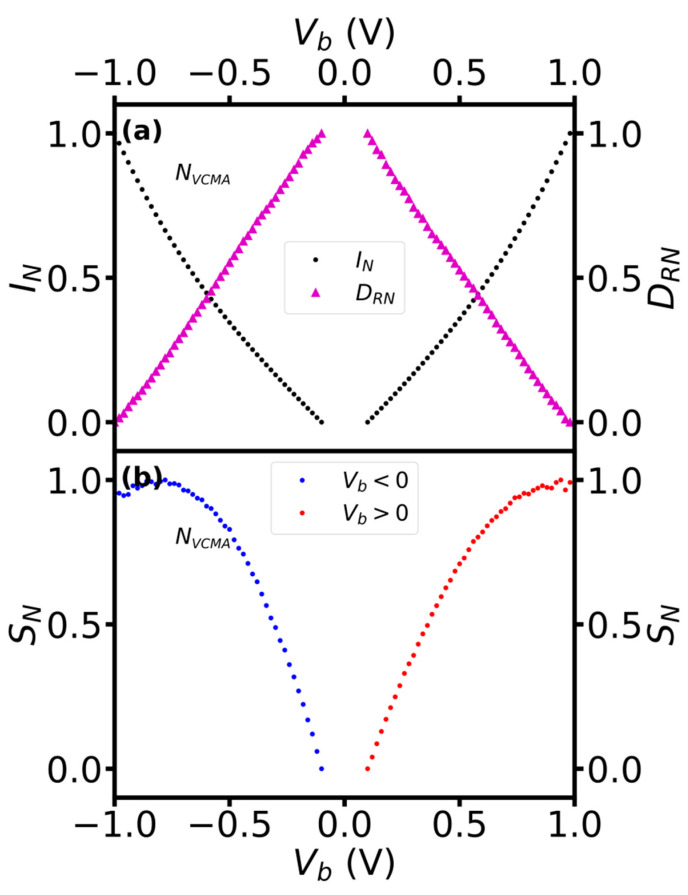
The normalized dependence of current (dots), derivative of R-H (triangles) (**a**) and sensitivity (**b**) on bias voltage of the sensors without VCMA modification.

The normalized dependence of the current and derivative of R-H on bias showed a crossover (Figure 7). We normalized the current and the derivative to evaluate their contributions to the bias dependence of the sensitivity. As expected, the current increases with bias, whereas the derivative reduces (Figure 7a). Their bias dependence showed, however, crossover at specific value (0.59 V) and approximately the same value for positive and negative bias. Moreover, the crossover appeared when the current and the derivative reached the normalized value of about (0.43). The crossover can be attributed as the cause of the typically observed saturation of the sensitivity with bias voltage in TMR sensors.

**Figure 8:**
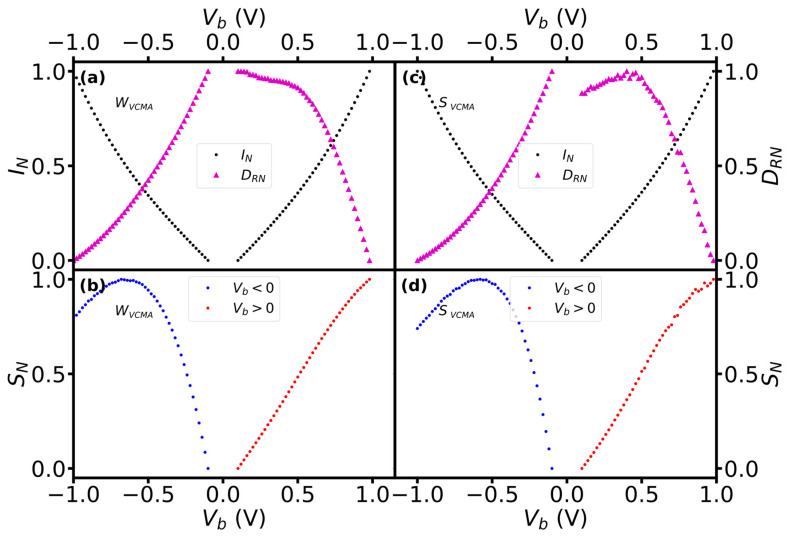
The normalized dependence of current, derivative of R-H and sensitivity on bias voltage for sensors with weak (**a**,**b**) and strong (**c**,**d**) VCMA modifications.

The crossover in the bias dependence of current and the derivative was also observed in sensors with VCMA modification, but at significantly higher voltage and current on positive than negative voltage (Figure 8). At a negative bias, the crossover appeared approximately at the same voltage bias (0.52 V) and normalized values of the current and the derivative values as for the sensors without VCMA modification (Figure 8a,c). At positive bias, however, the crossover appeared at a higher bias (0.73 V) and when the current reached as high as 0.62 of its maximal value. Moreover, the values of the current and voltage at which the crossover appeared were approximately the same for sensors with weak and strong VCMA modifications.

## 4. Conclusions

We investigated the dependence of sensitivity, derivative of R-H and current on the bias voltage of TMR sensors with and without VCMA modification. For the investigation, we fabricated sensors with different sensing layer thickness that resulted in the absence and presence of different degrees of the VCMA modification. The bias dependence of sensitivity, derivative of R-H and current were extracted from measured resistance versus magnetic field curves.

As expected, the sensor with no measurable VCMA modification showed typical [7,12] bias voltage dependence of sensitivity. The sensitivity increased at low bias voltage, it reached a maximum and then decreased. It was also not significantly affected by the bias polarity. Moreover, the sensors showed a monotonic decrease in dR/dμ_0_H and an increase in the current with bias. This concurrent reduction and increase in dR/dμ_0_H and current are responsible for the typical, as for standard design TMR sensors, dependence of the sensitivity on the bias voltage.

The sensors showing VCMA modification revealed new features in the bias dependence of sensitivity that were caused by the bias dependence of the derivative of R-H. At a bias polarity that reduces the VCMA strength, the sensitivity increases strongly without saturation up the measured range. This was because the current was increasing at a rate proportional to V^2^, and the derivative (dR/dμ_0_H) was increasing or remaining constant (depending on VCMA modification strength) up to a significant bias voltage. Moreover, even though the derivative at higher voltage started to reduce, the sensitivity was increasing due to the faster rate of the current increasing than reduction of the derivative. In contrast, at a bias polarity that enhances the strength of VCMA modification, the sensitivity remained approximately constant over a significant range. This was because the current was increasing at a rate proportional to V^2^ but the dR/dμ_0_H was reducing in the whole bias range. Thus, as a result the sensitivity remained effectively constant and reduced slightly only at higher bias.

This strong impact of bias polarity on the sensitivity change with bias magnitude resulted in high (up to 6.71) asymmetry of the sensitivity with respect to bias polarity. Moreover, the unusual dependence of the derivative of R-H on bias voltage was responsible for the observed increase in sensitivity without saturation for one polarity. Both I–V and dI/dV characteristics evidence a negligible influence of the bias polarity (VCMA modification) on the current change with bias in all sensors.

Sensors with and without VCMA modification showed the crossover in the bias dependence of current and derivative of R-H. Importantly, in all sensors, current dominated the bias dependence of sensitivity below the crossover voltage and above the derivative of R-H. Furthermore, the crossover voltage was unaffected by polarity in sensors without VCMA, whereas in sensors with VCMA, it appeared at a significantly higher voltage under positive polarity than negative polarity.

We used sensors with perpendicular anisotropy in the sensing layer. Therefore, future research is needed to confirm the crossover and the change in dominance contribution of current and the derivative to bias dependence of sensitivity for other TMR sensors that are based on other designs for linearization, such as external field biasing, weakly pinned sensing layers and superparamagnetic sensing layers [16].

## Data Availability

Not applicable.
